# Multimodal Environmental Sensing Using AI & IoT Solutions: A Cognitive Sound Analysis Perspective

**DOI:** 10.3390/s24092755

**Published:** 2024-04-26

**Authors:** Alexandros Emvoliadis, Nikolaos Vryzas, Marina-Eirini Stamatiadou, Lazaros Vrysis, Charalampos Dimoulas

**Affiliations:** Multidisciplinary Media & Mediated Communication Research Group (M3C), Aristotle University, 54636 Thessaloniki, Greece; nvryzas@jour.auth.gr (N.V.); mstamat@auth.gr (M.-E.S.); lvrysis@auth.gr (L.V.); babis@jour.auth.gr (C.D.)

**Keywords:** deep learning, environmental sound classification, audio encoding, Internet of Things, multi-modal sensing, resource-constrained environments, environmental monitoring

## Abstract

This study presents a novel audio compression technique, tailored for environmental monitoring within multi-modal data processing pipelines. Considering the crucial role that audio data play in environmental evaluations, particularly in contexts with extreme resource limitations, our strategy substantially decreases bit rates to facilitate efficient data transfer and storage. This is accomplished without undermining the accuracy necessary for trustworthy air pollution analysis while simultaneously minimizing processing expenses. More specifically, our approach fuses a Deep-Learning-based model, optimized for edge devices, along with a conventional coding schema for audio compression. Once transmitted to the cloud, the compressed data undergo a decoding process, leveraging vast cloud computing resources for accurate reconstruction and classification. The experimental results indicate that our approach leads to a relatively minor decrease in accuracy, even at notably low bit rates, and demonstrates strong robustness in identifying data from labels not included in our training dataset.

## 1. Introduction

The integration of Internet of Things (IoT) and Artificial Intelligence (AI) technologies into environmental monitoring has proclaimed a new era, transforming traditional approaches regarding smart city development and smart agriculture while enhancing sustainability and efficiency. In environmental monitoring, Smart Environment Monitoring (SEM) systems utilize IoT and modern sensors, along with machine learning techniques, for precise monitoring and effective management of both air and water quality, radiation pollution, and agricultural conditions [1]. For smart cities, IoT and AI technologies optimize urban operations, improving sustainability, productivity, and quality of life by analyzing extensive data generated from interconnected devices [2]. Additionally, this synergy plays a crucial role in developing environmentally sustainable smart cities, leveraging data-driven technologies alongside green strategies, to address urban environmental sustainability challenges [3]. In agriculture, the AIoT (convergence of AI and IoT) revolutionizes traditional farming practices by addressing key challenges such as pest management and post-harvest issues, making agriculture more efficient and resilient [4].

Building on these advancements, recent research has further validated the strengths and has addressed the limitations of sensor technologies integral to these systems. For instance, studies focusing on urban air quality monitoring in Lisbon [5] have revealed the capacity of sensor technologies to pinpoint pollution sources with high precision, confirming the crucial role of human activity in shaping environmental health [6,7,8]. Similarly, innovative deployments underscore both the adaptability and potential of sensor technologies to gather high-quality data across various domains, e.g., healthcare [9]. However, the deployment and maintenance of these technologies pose challenges, notably with regard to calibration and data integrity, necessitating frequent calibration and robust validation processes to ensure reliability [10]. Despite these limitations, the evolving landscape of sensor technology, supported by rigorous research, continues to drive progress in IoT and AI applications, promising more sustainable and efficient solutions across environmental monitoring, smart cities, and agriculture.

Incorporating mobile sensor technologies into IoT- and AI-powered systems can engage citizens in the process of data collection. Initiatives in IoT applications have pivoted towards enhancing community engagement [11] in tracking air pollution, introducing a model where citizens, armed with affordable sensor technology, play a crucial role in the data collection process [12]. This model not only democratizes the monitoring of air quality, but also boosts the diversity of the data collected, covering a wider geographic area and capturing more frequent updates. To further boost participation, the introduction of gamification techniques has been explored, leveraging the motivating aspects seen in applications within the Agri-Food sector focused on sustainability [13]. Fostering inclusive participation ensures that these environmental monitoring solutions are designed to be accessible and engaging for the entire community, while highlighting a comprehensive approach that merges technology with community action to promote environmental awareness and behavioral change.

As far as air pollution and environmental monitoring are concerned, extensive research to understand the relationship between air pollution and urban environmental factors has been conducted. This has resulted in the development of methods that utilize Deep Neural Networks (DNN) and Temporal Feature Integration (TFI) for accurate predictions regarding the levels of air pollution [14]. Current approaches consider various data modalities with air pollution information [15,16,17]. However, there is a noticeable gap in the collection and joint analysis of environmental audiovisual content in natural settings alongside air pollution data [18], or in combining such data with diverse modalities. Recent advancements in environmental sound classification have shown promising results. These methods use varied audio representations and DNN architectures, demonstrating their effectiveness in classification tasks. For example, ref. [19] extracts air pollution information through audio analysis. This process gathers audio through smartphones and conducts the environmental sound classification by identifying sources of pollution. The Bee-Mate module [20] has been implemented in mobile citizen science applications in order to address issues such as sensor calibration, data enhancement, citizen engagement, and gamification. This module allows citizens to capture audio–visual content with their smartphones. In order to identify the level of pollution for specific locations, it further processes this multi-modal information by engaging large audiences and capturing information for multiple sites. Bee-Mate exploits the potential of a DNN-based image classifier and the aforementioned sound classifier to conduct air pollution analysis.

However, these models often consist of a large number of parameters [21], posing a challenge in terms of implementation in edge devices due to their complexity and size. Moreover, and specifically for audio processing models, they usually receive spectral representations as inputs (i.e., spectrograms, mel spectrograms, etc.) [22]. This process not only adds overhead with respect to memory consumption, but also increases the processing costs. Therefore, instead of performing on-device downstream tasks, information can be gathered through edge devices and transferred to the resourceful cloud. Approaches such as those described in [23,24] are used to conduct on-device data gathering and transfer this information to powerful computational systems in order to perform downstream tasks. The current research focuses on increasing the feasibility and efficiency of audio-driven (indirect) air pollution monitoring (causing zero discomfort), thus making it available to broad audiences through citizen science models, with minimum intervention in terms of equipment and human effort requirements.

**Figure 1:**
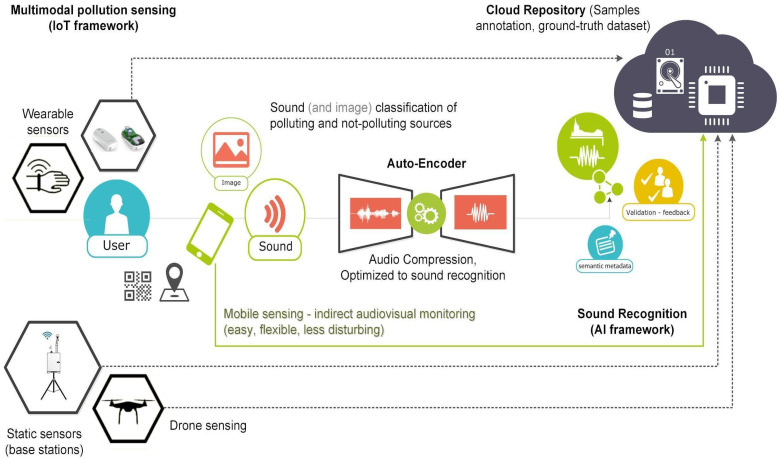
High-level representation of our proposed approach. Edge devices of an IoT network gather data, perform on-device coding, and transmit the information to the cloud. Powerful machines decode the gathered information and exploit large DNN-based models for downstream tasks.

In this study, we propose a system to efficiently transmit and classify urban environmental audio samples, as depicted in Figure 1. Within this context, the system receives audio through smartphones and encodes it into a smaller representation. This representation is transferred to the cloud, where it is decoded so that robust DNN-based classifiers conduct environmental analysis. The key contributions of this study are as follows:

- A DNN-based model is used to encode audio. The result of this process undergoes further encoding/decoding by exploiting traditional lossy and contemporary lossless encoding techniques.
- The compression method employs a minimal number of parameters, facilitating its implementation on devices with limited processing capabilities.
- The system is optimized for extremely low bit rate transmission to the cloud.
- The process allows for various classification tasks to be performed with the encoded and decoded audio, without any significant loss in accuracy.

In addition to enhancing citizen engagement in data collection processes, our proposed solution offers notable advantages for a wide range of applications that process audio data. By optimizing the encoding and transmission of audio samples, our approach not only minimizes associated costs, but also substantially reduces the storage requirements for maintaining this information. This optimization has far-reaching implications, potentially transforming practices in various sectors, such as traffic monitoring and industrial monitoring to name a few. Consequently, the broader adoption of our solution could lead to substantial cost savings and efficiency improvements across these diverse fields.

The paper is structured as follows. In Section 2, related work on environmental data analysis is referenced, in Section 3 the methodology, dataset preparation, architecture, and technical details of the proposed method are introduced, in Section 4 the experimental results are presented, in Section 5 the results are discussed and analyzed, and, in Section 6, the research is summarized and concluded.

## 2. Related Work

Since our method proposes a system that combines audio encoding and classification, it was considered essential that the current status of the literature concerning both scientific fields should be presented. Classification in the context of machine learning is a process where a model is trained to categorize data into predefined classes or labels. This is achieved by learning from a dataset that contains examples of different categories. The model, typically a form of a neural network or a statistical algorithm, learns to recognize patterns or characteristics that are indicative of each class. Once trained, the model can then be used to classify new, unseen data, assigning them to one of the learned categories. This technique is widely used in various fields, from image and speech recognition to medical diagnosis, where it aids in identifying and categorizing data based on learned patterns, thereby facilitating decision-making processes and predictive analyses.

Ref. [25] employed a technique that merged a substantial volume of unlabeled audio and visual data to generate embeddings, which were then used to train a classifier on a minimal dataset of labeled samples. This method integrates L3 embeddings [26] into a single vector using the x-vector approach [27]. This not only categorizes environmental sounds, but also identifies samples outside this category. Another study [28] devised a strategy for multi-channel audio analysis. This method uses raw, harmonic, and percussive log-mel spectrogram features and leverages models pre-trained on ImageNet for feature extraction in environmental sound classification. Ref. [29] introduced an approach that employs a pre-trained network for end-to-end audio embeddings generation through raw audio data. They also used transfer learning techniques for audio classification. Lastly, Ref. [30] utilizes a vision transformer pre-trained on ImageNet, applying transfer learning to environmental sound classification. In this approach, the raw audio input is transformed into a spectrogram representation by computing log mel-filterbank features. Specifically for environmental sound classification on edge devices, ACDNet [31] has been proposed as a lightweight DNN-based model that receives input raw audio, minimizing processing costs and memory consumption. The model is composed of two blocks: the Spectral Features Extraction Block (SFEB) and the Temporal Features Extraction Block (TFEB). Authors of ACDNet have also proposed a pipeline for compressing the model to fit in extremely resource-constrained environments, with a relative accuracy drop of 7%, while the relative drop in parameters is ~97.2%.

On the other hand, most DNN-based architectures receive a processed representation of the raw audio, usually in the form of spectrograms or mel-spectrograms, as input. Apart from increasing the processing costs, this step relies on larger audio chunks (5–10 s long) [32,33,34] and produces a combination of spectro-temporal representations with different window lengths and hop sizes, further increasing memory consumption. On top of that, the aforementioned methods rely on a vast number of parameters to provide high accuracy [31], representing a constraint on edge devices. Specifically, for applications that rely on multiple modalities for decision making (e.g., audio–visual content as in L3), the high computational demand and memory requirements can significantly hinder the deployment of these DNN-based systems. This is particularly challenging in scenarios where real-time processing is crucial, such as in interactive applications or those requiring immediate response. The integration of multiple data streams, such as audio and visual inputs, not only compounds the computational load but also necessitates sophisticated algorithms capable of efficiently synchronizing and interpreting these diverse data types. Consequently, there is a growing need for optimized models that balance accuracy with computational efficiency, especially for use in edge computing devices where resources are limited. Specifically, for IoT-based applications, Stamatiadou et al. [19] proposed a solution that involves a 1-D CNN-based classifier that reveals the relation of the gathered audio with air pollution. This system involves 1 s long audio chunks sampled at 22.05 kHz. Combined with the simplistic classifier, the proposed system does not increase processing costs nor memory consumption. 

Compression [35] involves the act of diminishing the size of a sample to make it more manageable for storage, transmission, or presentation. Compression algorithms fall into two primary categories: lossless and lossy. Lossless algorithms are capable of compressing and reconstructing the original sequence without any errors, whereas lossy algorithms introduce errors during the reconstruction process. Perceptual audio coding endeavors to blend elements of both lossless and lossy techniques, taking into account human perception to minimize the impact of information loss.

Advances in Artificial Intelligence have shown the potential of Neural Networks toward compression and reconstruction. Currently, developed speech-based DNNs compress the original audio while maintaining the overall quality on significantly low bit rates. The employment of loss functions based on psychoacoustic models [36] or based on the utilization of GAN-based architectures [37] has been proven capable of producing content distortion imperceptible to the human ear. Nevertheless, they typically comprise millions of parameters, presenting difficulties with respect to deployment in resource-constrained environments. With regard to audio coding on edge devices, Emvoliadis et al. [38] proposed a system that gathers environmental audio on edge devices that are equipped with a lightweight audio encoding method. The processed information is transferred to the resourceful cloud to reconstruct the original signal and perform classification. This work is built upon this system and extends it by minimizing the achieved bit rates using a two-stage audio coding scheme. The transferred information is either classified as it is or it is used to reconstruct the original signal. Finally, the method exploits an ensemble of pre-trained Computer Vision (CV) models [31] to perform multi-label classification.

Deep Learning has seen successful applications in the realms of audio compression and classification, yet there remains a notable void in fine-tuning audio encoding techniques for subsequent analytical tasks. Traditional and modern approaches to audio encoding have been tailored, predominantly, to human listeners, diverging from the needs of automated machine-learning-based audio classification. Our study seeks to fill this void by introducing a method specifically designed to encode and decode audio tailored to machine analysis rather than human ears. While current solutions for audio encoding rely on Deep Neural Network (DNN) architectures characterized by their extensive parameter counts, our approach utilizes a streamlined DNN model for the encoding and decoding processes. This model is then integrated with a traditional data compression scheme to achieve even greater compression rates, marking a significant advancement in the field.

## 3. Materials and Methods

This section describes the two-stage environmental sound compression and classification method. Each module is trained separately upon the same dataset, using different augmentation methods to increase the amount of training data. The proposed method is split into three mechanisms: data preparation, sound encoding–decoding, and sound classification mechanism.

### 3.1. Data Preparation

This subsection describes in detail the strategy for data gathering and preparation. Since our method refers to environmental monitoring applications, we exploited the ESC-50 [39] dataset, a labeled collection of 2000 audio chunks of 5 s each. These samples are organized into 50 semantic classes, each consisting of 40 samples. These classes are loosely arranged into five major categories:Animals, Natural Sounds, Human non-speech sound, Interior/domestic sounds, and Exterior/urban noises. From this collection, we defined as classes of interest the 22 that refer to urban environmental audio [34]. From these, we formed a binary taxonomy that generated distinct classes of sounds that are Pollution-Related (PR) and Non-Pollution Related (NPR). 

For the final dataset, we performed downsampling from 44.1 kHz to 22.05 kHz in order to decrease memory consumption costs in real-world scenarios. To avoid further increase in processing costs, the input to each model consisted of the raw audio waveform instead of the frequently used spectral representations. In addition, we exploited the PERSA framework [40] as a data preprocessing step in order to remove noisy and silent segments. Finally, we introduced a sliding window technique for data augmentation, with a window length of 25 ms, applied to each sample in the aforementioned dataset.

### 3.2. Sound Encoding–Decoding Mechanism

This subsection describes the sound encoding–decoding mechanism. As previously said, this mechanism is composed of a DNN model along with a conventional audio coding methodology, leading to a two-stage audio coding pipeline. Hence, this methodology is split into the DNN-based encoding and the conventional coding.

The Auto-Encoders (AEs) are commonly used algorithms from the field of Deep Learning (DL) for data compression that are able to capture complex structures. AEs are composed of two sub-networks, jointly trained: the encoder and the decoder. The encoder compresses the input sequence into a latent vector and the decoder, given this vector, reconstructs the original sequence. AEs are lossy algorithms, meaning that the reconstruction step will produce a sequence very close to the original one, yet not identical. Our proposed methodology fuses an AE with traditional compression schemas based on both lossy and lossless compression techniques, forming a two-stage compression and decompression approach. The first stage produces a compressed sequence via the encoder. Conventional compression schemas are applied to this output for further compression. The inverse process is followed to reconstruct the original signal.

#### 3.2.1. DNN-Based Encoding

AEs and their variations [41,42] have been widely studied in fields such as denoising, detection of anomalies, and data compression [43,44,45]. The main focus of this paper is data compression. Considering an input vector x of length L, AE compresses and reconstructs the input with the following operations.

(1) $$z_{c}=h(x;\vartheta_{E})$$

(2) $$y=g(z_{c};\vartheta_{D})$$

In Equations (1) and (2), the variable $z_{c}$ signifies the compressed representation resulting from the encoder. The symbols $\vartheta_{E}$ and $\vartheta_{D}$ represent the parameters linked to the encoder and the decoder, respectively. The functions h and g stand for the encoding and decoding operations. Additionally, x and y correspond to the original and reconstructed signals, respectively. For our experiments, we considered a three-convolutional-layer encoder, a dense layer to form the compressed representation, and a three-Transposed-Convolutional (TC)-layer decoder.

**Convolutional layer:** each convolutional layer was configured with a kernel of size 7 and had 32 k filters, where k represents the number of layers. Bias was introduced and a dropout layer (*p* = 0.2) followed to avoid overfitting. We employed the hyperbolic tangent (tanh) as the activation function to ensure that the output of each block contained both positive and negative values similar to the network input. Finally, downsampling was performed via average pooling layers.

**Dense layer:** the compressed representation was produced using a dense layer, which gets, as input, an $x_{in}\in \;R^{N\times L_{c}}$ tensor and, as output, a $x_{out}\in \;R^{1\times L_{c}}$. Therefore, the result was a non-linear mixing of the N filter outputs. This one-dimensional representation can be further compressed by an ordinary audio compression scheme formulated by lossy and lossless methods which will be described shortly.

**TC layer:** the compressed representation was fed into the decoder that consisted of TC layers. Each layer was configured so that its output matched the input of the mirrored encoder layer. Upsampling was performed by setting the stride of each TC layer to 2, while the activation function remained the same as in the convolutional layers and a dropout layer followed.

The abovementioned pipeline achieved a compression ratio of $F_{s}\times T\times L_{c}$, where $F_{s}$ is the sampling frequency of the original input, T corresponds to the signal duration in seconds, and $L_{c}$ is the length of the bottleneck representation. The decoder received the bottleneck output and was dedicated to generating a sequence nearly identical to the encoder input. The model was trained in the PR class. The data augmentation step involved a sliding window technique of 20 ms. The model was trained for 70 epochs using Adam optimizer with a learning rate of 0.0001, minimizing the Mean Squared Error (MSE) between original and reconstructed signals.

#### 3.2.2. Conventional Coding

The second stage compression employed a transformation to obtain the signal frequency components. These components were quantized and transformed into a byte sequence. The latter was further compressed using lossless compression schemas. As a first step, we applied a Discrete Cosine Transformation (DCT) [46] to the bottleneck output. DCT is a mathematical transformation, formulated in Equation (3), that analyzes signals with respect to their frequency components and is commonly used for audiovisual content compression. The result of this transformation underwent quantization via multiplication with $2^{N}$, where N represents the quantization level, and was then rounded down to the nearest integer. This series of steps generated a sequence of integer values. Subsequently, we created a byte-based representation of the integer sequence. This aimed at leveraging state-of-the-art lossless compression algorithms. Lossless compression identifies and exploits repetitions or similarities and patterns present in the original sequence. An example of a traditional lossless compression algorithm is Lempel–Ziv–Welch (LZW) [47]:

(3) $$X_{k}=\sum_{n=0}^{N-1}x_{n}cos\left[\pi /N(n+0.5)k\right],\;k=0,1,\ldots ,N$$

LZW replaces repetitive data with references to earlier instances of the same data. It employs a fixed-length sliding window and a lookahead buffer to scan input data. At each buffer position, it searches for the longest matching sequence within the sliding window. It then encodes the pair (L, D), where L represents the match length and D is the backward offset from the current position to the start of the matched data in the sliding window. During decoding, a sliding window and a buffer store the decompressed sequence. If a match is found, the algorithm reads and transfers data from the sliding window, otherwise it copies a literal character.

Brotli [48] and Zstandard (Zstd) [49] are SOTA lossless compression algorithms developed by Google and Facebook, respectively. Brotli combines LZW, Huffman coding, and second-order context modeling [50], leading to faster encoding and improved compression ratios than traditional methods. Brotli utilizes a predefined dictionary of 120 kB in size. Zstd also relies on the LZW approach and combines it with finite state entropy [51] and Huffman. Similar to Brotli, Zstd uses a dictionary-based approach. Lastly, Zstd provides a learning dictionary method that efficiently compresses various data types. In our proposed approach, unlike VQ-VAE [41] which relies on a predefined dictionary and increases memory consumption, both Brotli and Zstd are able to dynamically encode data without the need for a pre-defined dictionary, thereby maintaining memory consumption at feasible levels.

**Figure 2:**
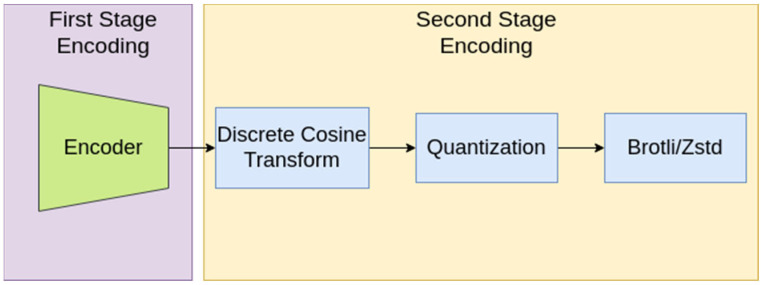
The two-stage encoding method. The encoder output is processed through DCT. The extracted frequency components are quantized and encoded by Brotli or Zstd.

The overall encoding pipeline is depicted in Figure 2. Captured audio was the input to our model. The encoder was applied as the first compression stage and was followed by DCT to obtain the backbone frequency components. This sequence, given a level, was quantized and floored. The output was transformed into a list of bytes that was further compressed via the aforementioned lossless compression algorithms in a Variable Bit Rate (VBR) fashion. For our experiments, we considered the DCT type II and the quantization levels range between 4 and 6.

### 3.3. Classification Mechanism

The ESC-50 dataset was processed to generate a new taxonomy that included sounds either related or not related to pollution. Since our approach involves audio processing in IoT-based applications, we also deal with the whole ESC-50 taxonomy and the super categories into which these classes are loosely arranged.

The former classification mechanism concerns the ACDNet. ACDNet has also been exploited in a pipeline for compressing and deploying the model into extremely resource-constrained environments. In addition, we exploited the classifier proposed in [34]. For this specific classifier, we considered two topologies: one that received the reconstructed signal, containing a larger number of parameters, and one that classified the encoded audio, which is composed of fewer parameters. Each classifier was trained as proposed in [30] for reproducibility purposes.

Regarding the latter classification mechanism, the outcome of the two-stage audio coding method was transferred to the cloud and was used to reconstruct the original signal. This information was utilized to generate spectral representations as in [31]. These representations were fused and served as input to an ensemble of fine-tuned CV pre-trained models. This scheme aimed to perform classification over the total classes and their super categories. The rationale behind this mechanism was to evaluate the potential of our method to examine whether it is possible to achieve extremely low bit rates regarding on-device audio coding and perform classification on the cloud without sacrificing accuracy. By this, we aimed to minimize the computational costs on the Device Layer and the transferring costs to the Server Layer. The latter is equipped with very large models that have shown their potential on heavy classification tasks, while their size is a major constraint when deploying them on low-cost sensors. The training process and hyper-parameters were the same as those described in [31].

### 3.4. Overall Pipeline

**Figure 3:**
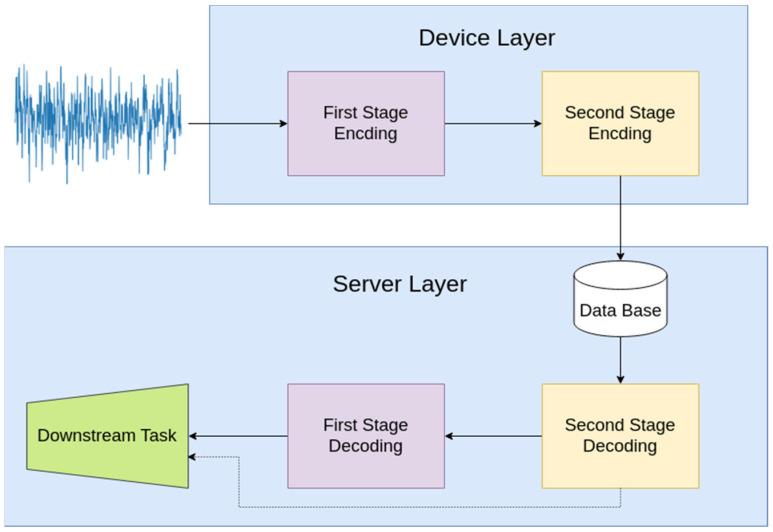
The proposed methodology. The Device Layer is equipped with the two-stage encoding algorithm. The output of this process is transmitted and decoded in the cloud. Downstream tasks could now be performed through large DNN-based models on the auto-encoder’s reconstructed latent vector (second-stage decoding) or the reconstructed signal.

The previous sections describe the proposed methodology. In real-world applications, the Device Layer is equipped with the AE encoder and the conventional encoding algorithm. This cascaded compression scheme achieves significantly low bit rates, indicating that the encoded audio can be easily transferred. Server Layer received the processed information and reconstructed the originally received audio. Either the reconstructed or the compressed signal can be used for downstream tasks. The overall pipeline is depicted in Figure 3.

## 4. Results

Due to the combination of an audio encoding–decoding process and a classification process that our approach exploits, the performance metrics should not only refer to the classification task, but also should consider the quality of the reconstructed signal. That is why we evaluate the potential of the proposed encoding–decoding scheme under essential metrics for signal reconstruction. The first sub-section provides information about the quality of the reconstructed signal utilizing objective metrics gathered from the literature. The second provides insights into the classification mechanisms. These refer to both binary and multi-label classification tasks. Binary classification involves the ACDNet and two conventional 1-D CNN classifiers, which receive as input either the reconstructed or the compressed signal. On the other hand, the multi-class classification task refers to the 50-class classification and the soundscape classification problem (five-class classification). These classification schemas involve an ensemble of pre-trained CV models, assuming that the encoder and its output can be deployed and transferred through resource-constrained environments. Finally, we have conducted five-fold cross-validation as suggested by the dataset’s developers.

### 4.1. Audio Reconstruction

**Figure 4:**
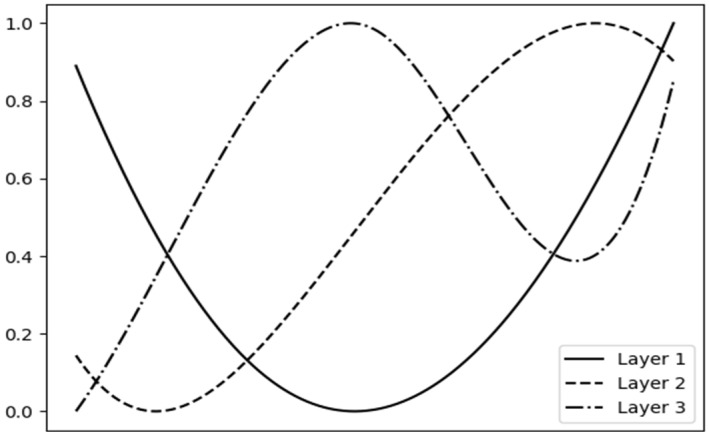
Time-domain representation of the filters that the DNN-based encoder developed during training. *Y*-axis denotes the magnitude of each filter. *X*-axis denotes the convolutional layers points.

**Figure 5:**
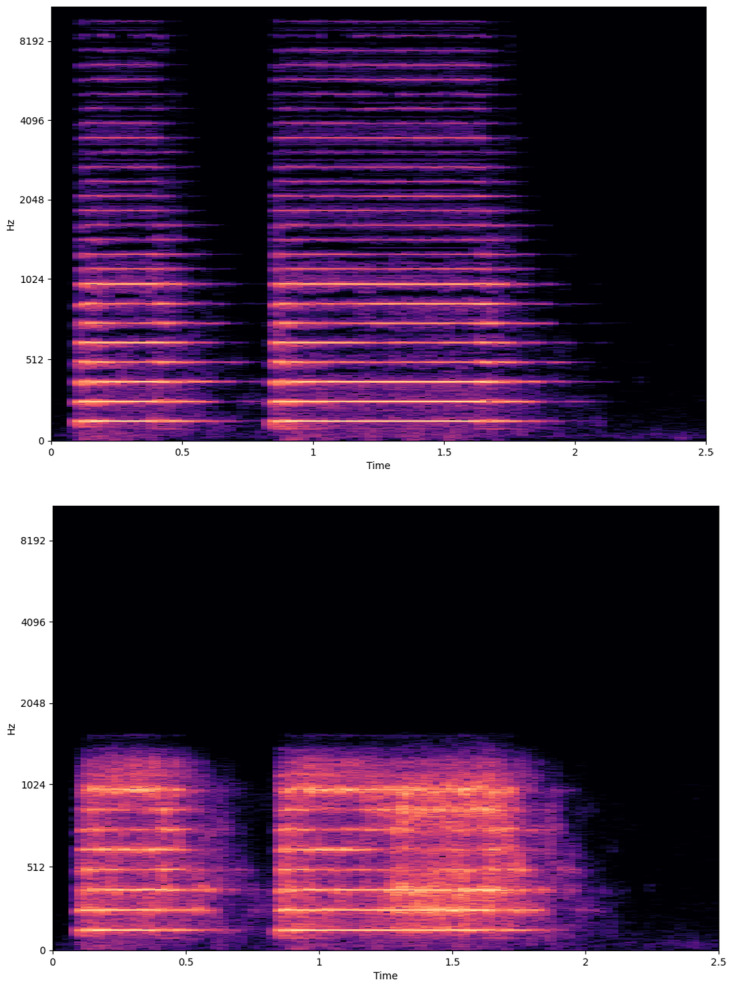

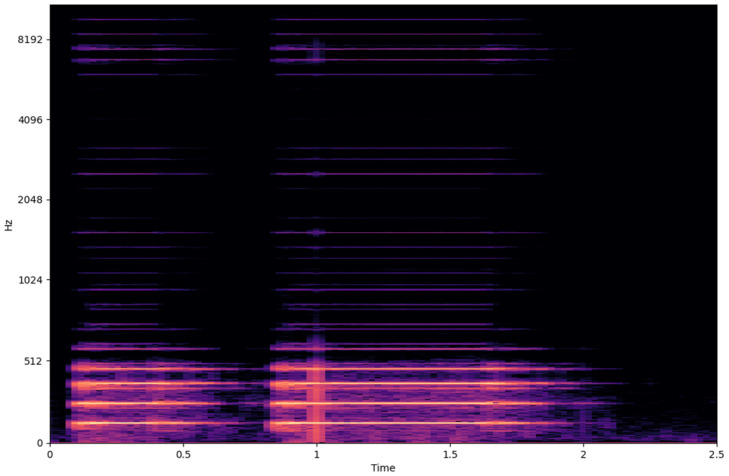
Examples of log-power spectrograms regarding the original (**top**), Opus operating at 6 kbps (**middle**), and the proposed codec (**bottom**) operating at 3.3 kbps (AE + Brotli, N = 4). Both Opus and the proposed method insert noise in low frequencies and cannot capture higher frequency components. On the other hand, Opus seems to be noisier than the proposed approach.

As discussed in Section 3.2.1, the utilized DNN model is relatively simple, incorporating convolutional and transposed convolutional layers. It is worth noting that the AE model was trained upon samples that exist in the PR class in order to evaluate its robustness on samples that do not exist in the training set (e.g., samples from the NPR class). The plain deep encoder received as input a vector of length 22,050 while outputting a vector of length 2751, leading to a compression ratio of ~8 utilizing approximately 50 k parameters. The final bit rate was computed by transforming the second stage output from a list of bytes to a sequence of bits. To investigate the representations learned by the deep encoder, we retrieved the time-domain filters produced during training (Figure 4). These filters reveal a functionality similar to that of low-pass filters. The first layer consists of parabolic filters, the second layer generates ramp-like filters, while the last layer introduces more complex, similar to triangular, filters. Each one of the developed filters generates an output with a decreased dynamic range. Examining the log-power spectrograms of the original and reconstructed audio (Figure 5), we observe that the backbone low-frequency characteristics are captured precisely. However, our method fails to capture high-frequency components and inserts clicks. This can be verified by examining the differences between the two spectrograms. While being unable to preserve high-frequency components, traditional audio codecs introduce noise in the low-frequency ones.

Here, we compare the proposed method against Opus [52] conventional codec. In order to assess the efficacy of the proposed approach, we measured the performance of three key metrics: Peak Signal to Noise Ratio (PSNR), Structural Similarity Index Metric (SSIM) [53], and Perceptual Evaluation of Audio Quality (PEAQ) [54]. To calculate PSNR, we extracted the squared dynamic range of the original signal and divided it by the Mean Squared Error (MSE) between the original and reconstructed signals. For SSIM and PEAQ, signal normalization within the range between 0 and 1 was executed. Specifically for PEAQ, we performed downsampling to 16 kHz to match the requirements of the used software. A comprehensive comparison between different configurations and Opus codec is presented in Table 1. There, it can be observed that the reconstructed signal slightly degraded from the AE output, even for significantly low bit rates. Both compression formats show similar performance, with Zstd being faster while Brotli achieved lower bit rates. Using a predetermined dictionary was not found to enhance performance, whereas employing an empty dictionary was found to prevent additional memory usage.

Finally, we evaluated our method’s performance on signal reconstruction with respect to the NPR class at significantly low bit rates. This experiment allowed us to examine the level of generalizability of the proposed method and the results are presented in Table 2.

### 4.2. Audio Classification

In this part of the experiment, we evaluated the performance of our approach regarding the classification task using frequently used classification metrics. Precision is defined as the ratio between the number of True Positives (TP) and the total number of TP and False Positives (FP). Recall is defined as the ratio between the number of TP and the total number of TP and False Negatives (FN). F1-score is an informative metric used for imbalanced classification problems and is defined as the harmonic mean between precision and recall. Finally, accuracy is defined as the ratio between the total number of TP and True Negatives (TN) and the total number of samples in the set.

#### 4.2.1. Binary Classification

For the binary classification task, at first, we examined the behavior of a SoA classifier and two 1-D CNN-based classifiers. For the latter, each classifier received the reconstructed and compressed signal, accordingly. This step allowed to:

(1) Examine the behavior of each classifier with respect to each representation.
(2) Conduct a fair comparison.

In addition, we accounted for the complexity of each classifier. ACDNet is composed of roughly 4.7 M parameters. On the other hand, the 1-D CNN classifier that receives the reconstructed and original signals includes ~100 k parameters and the classifier that receives the compressed representation as input includes ~80 k parameters. Table 3 summarizes the classification results regarding each classifier and each representation regarding our pipeline.

From Table 3, it can be observed that ACDNet outperformed any other model with respect to the binary classification setup. The 1D-CNN that received the original input showed poor performance as it performed slightly better than a random classifier. On the other hand, there was a relatively low drop in terms of relative accuracy with respect to the ACDNet for both original and reconstructed signals. The conventional 1D-CNN model showed a better performance when receiving the reconstructed signal as input instead of the original audio. Lastly, the compressed representation was found to be more easily classified by such a model, compared to the original and reconstructed signals.

The above results refer to classification mechanisms that received the original and compressed or reconstructed signals that the plain AE had generated. Following introduction of the second stage audio coding scheme, the bit rate experienced a reduction, while the produced signal did not degrade a lot compared to the reconstructed signal that the plain AE had produced, as Table 1 presents. Given these results, we further exploited the ACDNet architecture along with different quantization levels to classify between PR and NPR classes, achieving significantly low bit rates. This task employed both Brotli and Zstd lossless algorithms to evaluate the potential of the proposed two-stage audio encoding–decoding methodology.

Table 4 shows that, while our approach was overtaken by Opus at low bit rates, it tended to experience a rapid decline in classification performance as the bit rate dropped. Conversely, our method demonstrated noteworthy performance, particularly at remarkably low bit rates, and, despite the absence of high-frequency harmonics, outperformed Opus when the latter operated at higher bit rates. Regarding the lossless state-of-the-art algorithms that our method employs, both revealed their robustness in terms of classification performance.

#### 4.2.2. Multi-Label Classification

As a final evaluation step, we followed the exact same approach proposed in [31], which is a viable solution for our set-up. At first, the encoder network was lightweight, consisting of less than 50 k parameters. Then, the achieved bit rates, the reconstructed signal’s distortion, and the performance of a state-of-the-art classifier showed that a machine is able to perform binary classification. 

Therefore, we processed the whole ESC-50 taxonomy under the proposed two-stage audio coding scheme and fine-tuned several pre-trained CV models. More specifically, this approach split each 5 s sample into 1 s segments and passed these through the proposed audio coding method. These segments were concatenated to form the original audio. We generated three mel-scaled spectrograms with varying hop lengths and window sizes, forming the input to each pre-trained CV model. For our experiments, we considered a DenseNet [55], a ResNet [56], a ConvNext [57], an EfficientNet [58] and a Wide ResNet [59]. 

After evaluating each model individually, we built an ensemble of these models and compared the results with a pre-trained ResNet-18, fine-tuned on the original audio samples, following the exact same process. Finally, this experiment considered the total of the 50-class classification and the five-class classification tasks.

By observing Table 5, it can be evinced that the ResNet-18 trained upon the original audio signals delivered the best performance with respect to both classification experiments. Models that received the reconstructed audio—encoded with significantly low bit rates—as input failed to provide accurate results, especially for the 50-class classification task. However, setting up an ensemble of models seemed to improve the performance. Specifically for the five-class classification task, the ensemble achieved a relative accuracy drop of 7.17 compared to the ReNet-18 that exploited the original audio. 

In addition, ResNet-18 has a size of 44.7 MB, while the encoding method of our proposed approach occupies 400kB of memory. The achieved bit rates reveal the potential of the proposed method in real-world applications that relate to relatively simple problems, such as the binary classification of PR and NPR and soundscape categorization. Nevertheless, the proposed approach failed to perform accurate classification in larger classification tasks, probably due to the limited parameters and the significantly low bit rates.

## 5. Discussion

To summarize, the proposed method exploits a DNN-based auto-encoder model in order to extract meaningful features for the classification task of environmental sounds. Moreover, a second-stage compression scheme that exploits a traditional lossy compression method (DCT) and a state-of-the-art lossless encoding algorithm (Brotli and Zstd) is introduced for further bit rate reduction. The overall pipeline has been validated against three classification tasks: a binary classification between sounds that are related to pollution and those that are not, a five-class classification task regarding the super categories defined in the ESC-50, and the overall 50-class classification. 

The suggested approach demonstrates that achieving very low bit rates (under 6 kbps) with minimal processing costs is feasible but accompanied by certain limitations. Specifically, at these very low bit rates, the approach tends to suppress high-frequency components while introducing noise in the lower frequencies. Additionally, for complex classification problems, such as the 50-class categorization of the ESC-50 dataset, the method suffers from a significant accuracy drop of about 10%.

**Figure 6:**
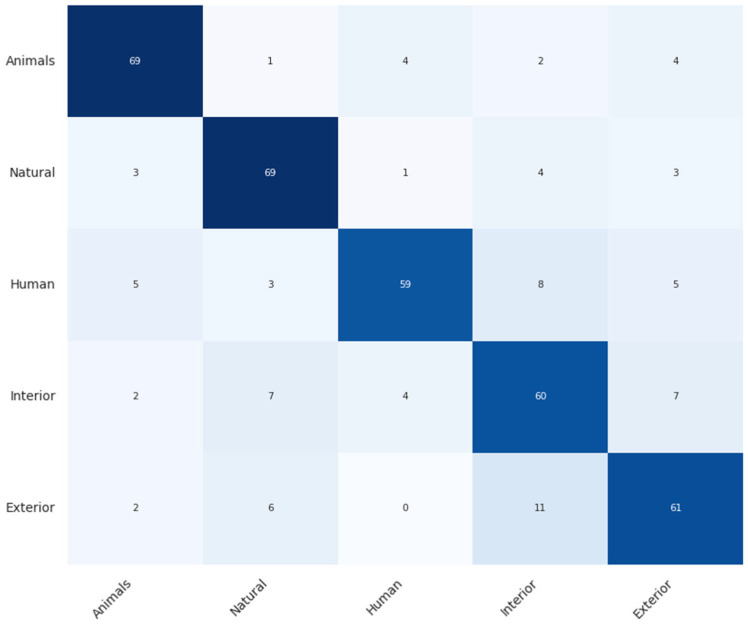
Confusion matrix relative to the five-class (super category) classification task.

However, it is important to note the relative performance advantages of this approach over alternatives such as Opus. Despite having similar challenges in frequency suppression and noise introduction, the suggested method outperforms Opus in terms of overall sound quality. Furthermore, for simpler classification tasks, such as binary and five-class (super categories of ESC-50) classifications, the performance of this approach does not significantly deviate from the performance of classification of the original sound, as presented in Figure 6. Impressively, across all three classification tasks examined, the proposed approach demonstrates superior performance compared to Opus, highlighting its potential in applications where low bit rate and efficient processing are crucial.

The proposed method encounters difficulties in handling complex classification tasks for three primary reasons. Firstly, the relatively small structure and limited number of parameters of the DNN-based encoder restrict its ability to capture meaningful features from complex soundscapes. This limitation, combined with the achieved extremely low bit rates, is particularly problematic for sounds that contain high-frequency components, such as those produced by birds and insects found in the ESC-50 dataset, which may suffer from poor reconstruction quality due to the suppression of high frequencies at very low bit rates. Additionally, to keep processing costs low, the method processes raw audio waveforms directly, foregoing the extraction of spectro-temporal representations that could allow for more detailed analysis in both time and frequency domains.

Despite these challenges, the method shows a robust capability for simpler classification tasks, as can be seen in Figure 6. Its low processing demands and minimal time delay make it well-suited for streaming applications. Moreover, by utilizing an encoding–decoding scheme, this approach facilitates the transfer of audio samples to the cloud for accurate classification and human supervision, circumventing the limitations of on-device classification. This setup not only ensures accurate classification and potential for human oversight, but also allows for the transmission of encoded samples at extremely low bit rates. Consequently, it reduces the memory required for storage and supports ongoing learning by accumulating and utilizing less space-intensive samples.

## 6. Conclusions and Future Work

This work proposes an audio encoding and classification process with applications in resource-constrained environments for multimodal data gathering. Using a scheme as such allows for the exploitation of large DNN-based models, as the encoded signal is effectively being transferred to the resourceful cloud. The proposed method overcomes processing costs and memory limitations, as the signal is encoded via significantly low bit rates. Additionally, the size of the encoding model is relatively small, indicating its ability to be deployed in resource-constrained environments. The gathered samples can be fused to fine-tune large pre-trained CV models that cannot be deployed in environments with limited resources. Moreover, these models usually require spectro-temporal representations that not only demand high processing power, but also increase memory consumption. Our method is able to effectively handle these obstacles by efficiently transferring data to the cloud.

The lightweight encoding process, along with the achieved bit rates, are strong indicators that the proposed methodology can be effectively deployed in pipelines that handle multimodal data. Furthermore, this method could be fused in data pipelines that gather and process visual data and environmental monitoring variables and lead to more accurate predictions or to the detection of anomalies.

While the classification model seems to be robust with respect to the binary classification task, this behavior is not observed in the five-class and 50-class classification tasks. The introduction of noise in the low-frequency regime and the suppression of high-frequency components seem to confuse the model. However, the proposed encoding–decoding process introduces less noise than Opus, while the fine-tuned CV classifiers perform better when it comes to samples produced through our proposed approach. Moreover, the achieved compression ratios and efficiency in terms of processing costs can be used in already developed systems for broadcasting audiovisual information [60] and within web-based applications that also rely on deep-learning-based processing systems [61].

In our future work, we will consider techniques that effectively compress DNN-based models. We will increase the number of parameters and perform adversarial training with psychoacoustic model-based losses, as proposed in recent studies. We will evaluate the potential of asymmetrical encoder–decoder architectures [62], while maintaining the number of encoder parameters, and apply a super-resolution-based decoder to improve the method’s performance regarding high-frequency components. As a final step, we will consider the deployment of our method in devices that gather information about the levels of pollution and fuse both modalities in pipelines that predict future pollution levels and perform joint analysis. Moreover, the same techniques will be evaluated for multimodal data, for images, video, and audiovisual information channels in an information fusion approach. While old approaches that exploit multichannel (spatial) audio encoding and processing use high-end dedicated equipment and network infrastructures [60], modern solutions exploit mobile devices with fewer capacities in audio-related problems, also employing distributed multimodal sensing supplemented with AI automations (e.g., multi-channel speaker localization and diarization, web TV indexing automations, etc.) [63]. Hence, the proposed approach and the achieved efficiency points to the direction of machine cognitive coding.

## Figures and Tables

**Table 1:** Evaluation results relative to the PR class regarding the quality of the reconstructed signal. * implies the usage of a predefined dictionary.

Method	Level (N)	Bit Rate (kbps)	PEAQ	SSIM	PSNR (dB)
AE	––	44	3.34	0.84	29.8
AE + Brotli *	6	8.9	3.26	0.83	28.9
	5	5.9	3.07	0.82	27.6
	4	3.3	2.79	0.80	25.5
AE + Brotli	6	8.9	3.26	0.83	28.9
	5	5.9	3.07	0.82	27.6
	4	3.3	2.79	0.80	25.5
AE + Zstd *	6	9.2	3.26	0.83	28.9
	5	6.1	3.07	0.82	27.6
	4	3.4	2.79	0.80	25.5
AE + Zstd	6	9.2	3.26	0.83	28.9
	5	6.1	3.07	0.82	27.6
	4	3.4	2.79	0.80	25.5
Opus	––	44	4.16	0.92	34.1
	––	12	3.26	0.80	28.6
	––	6	2.41	0.68	24.7

**Table 2:** Evaluation results relative to the NPR class regarding the quality of the reconstructed signal at significantly low bit rates.

Method	Level (N)	Bit Rate (kbps)	PEAQ	SSIM	PSNR (dB)
AE	––	44	3.08	0.82	29.0
AE + Brotli	4	2.6	2.58	0.79	25.0
AE + Zstd	4	2.8	2.58	0.79	25.0
Opus	4	6	2.46	0.70	24.7

**Table 3:** Classification results regarding the original and reconstructed representations of the plain AE for different classification models. Names in parentheses indicate the input to each model.

Model	Precision	Recall	F1-Score	Accuracy
ACDNet (original)	90.04% (4.01)	90.27% (3.27)	90.24% (3.34)	91.47% (4.34)
1D-CNN (original)	73.27% (6.52)	74.42% (5.86)	73.63% (5.12)	74.25% (5.16)
ACDNet (reconstructed)	89.00% (6.23)	89.81% (4.62)	88.87% (1.53)	89.25% (1.69)
1D-CNN (reconstructed)	81.77% (8.68)	82.26% (6.12)	80.63% (5.34)	80.75% (5.16)
1D-CNN (compressed)	86.22% (4.32)	86.07% (3.73)	85.91% (2.56)	85.75% (2.91)

**Table 4:** Classification results of different audio coding configurations and the original audio that the ACDNet classifier received as input.

Input	Level (N)	Bit Rate (kbps)	Accuracy
Original	––	256	91.47%
AE (plain)	––	44	89.25%
AE + Brotli	4	3.3	87.97%
	5	5.9	88.65%
	6	8.9	90.88%
AE + Zstd	4	3.4	87.97%
	5	6.1	88.65%
	6	9.2	90.88%
Opus	––	44	89.75%
	––	12	86.52%
	––	6	72.34%

**Table 5:** Classification results with respect to pre-trained CV models. ResNet-18 was fine-tuned on the original audio, while other models were fine-tuned using our method with Brotli at N = 4. The 50-class refers to the total ESC-50 classification. The five-class refers to ESC-50’s super categories (Animals, Natural Sounds, Human non-speech sound, Interior/domestic sounds and Exterior/urban noises).

Model	50-Class Accuracy	5-Class Accuracy
ResNet-18 (original)	84.25% (2.70)	89.85% (1.65)
ConvNext (3.3 kbps)	73.90% (3.28)	82.20% (4.19)
ResNet-50 (3.3 kbps)	73.20% (4.01)	81.75% (3.85)
DenseNet-101 (3.3 kbps)	73.15% (3.59)	82.70% (3.92)
Wide ResNet (3.3 kbps)	73.40% (3.22)	82.60% (4.06)
EfficientNet (3.3 kbps)	73.70% (3.12)	82.10% (3.28)
Ensemble (3.3 kbps)	74.60% (3.06)	83.40% (3.14)

## Data Availability

Data are contained within the article.
